# A 1.6-Mb Microdeletion in Chromosome 17q22 Leads to *NOG*-Related Symphalangism Spectrum Disorder without Intellectual Disability

**DOI:** 10.1371/journal.pone.0120816

**Published:** 2015-03-27

**Authors:** Xiuhong Pang, Huajie Luo, Yongchuan Chai, Xiaowen Wang, Lianhua Sun, Longxia He, Penghui Chen, Hao Wu, Tao Yang

**Affiliations:** 1 Department of Otolaryngology-Head and Neck Surgery, Xinhua Hospital, Shanghai Jiaotong University School of Medicine, Shanghai, China; 2 Ear Institute, Shanghai Jiaotong University School of Medicine, Shanghai, China; 3 Shanghai Key Laboratory of Translational Medicine on Ear and Nose Diseases, Shanghai, China; 4 Department of Otorhinolaryngology-Head and Neck Surgery, Taizhou People’s Hospital, Jiangsu Province, China; 5 Department of Otolaryngology, Renji hospital, Shanghai Jiaotong University School of Medicine, Shanghai, China; CNRS UMR7275, FRANCE

## Abstract

Microdeletions in chromosome 17q22, where the *NOG* gene resides, have been reported leading to the *NOG*-related symphalangism spectrum disorder (*NOG*-SSD), intellectual disability and other developmental abnormalities. In this study we reported a dominant Chinese Han family segregating with typical *NOG*-SSD symptoms including proximal symphalangism, conductive hearing loss, amblyopia and strabismus, but not intellectual disability. Sanger sequencing identified no pathogenic mutation in the coding regions of candidate genes *NOG*, *GDF5* and *FGF9*. SNP genotyping in the genomic region surrounding *NOG* identified loss of heterozygosity in the affected family members. By array comparative genomic hybridization and quantitative real-time polymerase chain reaction, we identified and mapped the breakpoints of a novel 1.6-Mb microdeletion in chromosome 17q22 that included *NOG* and twelve other genes. It is the first microdeletion reported in chromosome 17q22 that is associated with *NOG*-SSD only but not with intellectual disability. Our results may help identifying the dosage sensitive genes for intellectual disability and other developmental abnormalities in chromosome 17q22. Our study also suggested that genomic deletions in chromosome 17q22 should be screened in the *NOG*-SSD patients in which no pathogenic mutation is identified by conventional sequencing methods.

## Introduction

*NOG* encodes noggin, the first identified bone morphogenetic protein (BMP) antagonist [1]. It plays an important role in proper bone and joint development [2]. Mutations in *NOG* may lead to a series of autosomal dominant disorders called the *NOG*-related symphalangism spectrum disorder (*NOG*-SSD). *NOG*-SSD is mainly characterized by symphalangism, the ankylosis of the joints in fingers or toes. Other symptoms of *NOG*-SSD may include conductive hearing impairment due to stapes ankylosis, fusion of the carpals and tarsals, brachydactyly, abnormal faces, hyperopia and strabismus. In addition to *NOG*, *GDF5* and *FGF9* are two other genes associated with *NOG*-SSD [3,4].

Genomic disorders are caused by complete loss, gain or disruption of one or multiple dosage sensitive genes. Over ten microdeletions in the chromosome 17q22 region, where *NOG* resides, have been reported causing *NOG*-SSD, intellectual disability (ID) and other developmental abnormalities in a dominant manner [5–10]. Those microdeletions range from 1.86 Mb to over 20 Mb and result in single-copy loss of ten to hundreds of genes. Though haploinsufficiency of *NOG* is clearly the key pathogenic mechanism for *NOG*-SSD, gene(s) in chromosome 17q22 that are directly involved in the etiology of ID and other developmental abnormalities remain elusive.

In the present study, we identified a novel 1.6-Mb microdeletion in chromosome 17q22 as the cause of *NOG*-SSD in a dominant Chinese Han family. The affected family members did not have ID or other developmental abnormalities commonly seen in patients with chromosome 17q22 microdeletions. Our results may provide new insights into the pathogenic mechanism for the ID associated with the chromosome 17q22 microdeletions.

## Materials and Methods

### Subjects and clinical examinations

**Fig 1:**
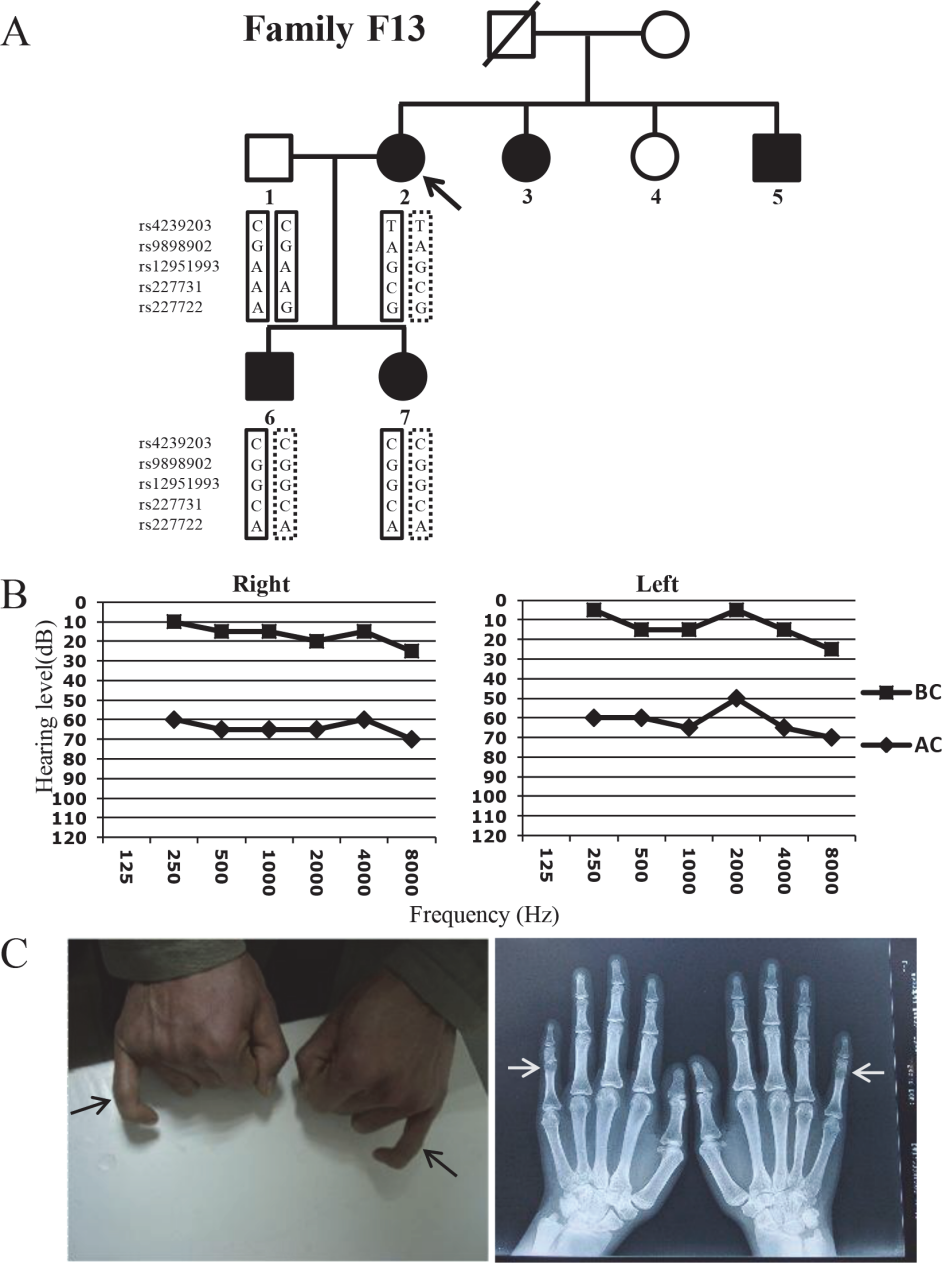
Pedigree, genotype and phenotype characterization of Family F13. A) Pedigree and SNP genotypes of Family F13. The proband F13-2 is pointed by the arrow. SNP genotypes of subjects F13-1, F13-2, F13-6 and F13-7 showed a loss of heterozygosity (in dotted box) in the affected individuals. B) Representative audiograms of subject F13-6. The gaps between the air- (AC) and bone-conducted (BC) hearing thresholds indicate a conductive hearing loss. C) Images and digital radiography of the hands of subject F13-5 showing fusion of the proximal interphalangeal joints at the fifth fingers (arrows).

Family F13 with *NOG*-SSD was recruited from Shanghai, China including five affected subjects and two unaffected subjects (Fig. 1A). A detailed physical examination was performed in all five affected subjects with special attentions to the possible skeletal, auditory, ophthalmologic and mental abnormalities. The conductive hearing loss was confirmed by the air- and bone-conduction pure-tone audiometry. The fusion of the proximal interphalangeal joints was confirmed by radiography. All subjects or their parents on behalf of the children gave written, informed consent to participate in this study. This study was approved by the Ethics Committee of the Shanghai Jiao tong University School of Medicine, Xinhua Hospital.

### Mutation screening by Sanger sequencing

Genomic DNA was extracted from the whole blood using the Blood DNA kit (TIANGEN BIOTECH, Beijing, China). Exons and flanking introns of *NOG*, *GDF5* and *FGF9* were PCR amplified and bi-directional sequenced as previously described [3,4,11].

### SNP genotyping

Five SNPs (rs4239203, rs9898902, rs12951993, rs227731 and rs227722) within 150 kb in distance to *NOG* were selected to identify the potential loss of heterozygosity (LOH) in the affected family members. The SNPs were genotyped by PCR amplification and Sanger sequencing.

### Array comparative genomic hybridization (array-CGH)

Array-CGH was performed for proband F13-2. Briefly, genomic DNA was digested, ligated, PCR amplified, labeled and hybridized to the Affymetrix Genome-Wide Human SNP Nsp/Sty 6.0 chip following the manufacture’s guide (Affymetrix, Santa Clara, CA, US). Arrays were scanned by GeneChip Scanner 3000 and analyzed by Command Console Software 3.1 with default settings. Raw data that passed the quality control were further analyzed by Genotyping Console Software to obtain genotype call of each SNP locus. SNP call rate of sample F13-2 was 94.67%.

### Real-time PCR

Real-time PCR was performed for proband F13-2 and a normal hearing control on the 7300 Realtime PCR System (Applied Biosystems) using SYBR Premix Ex Taq (Takara Bio Company). The primers were designed by the PRIME3 software online (http://frodo.wi.mit.edu/cgi-bin/primer3/primer3_www.cgi). Each reaction was repeated tree times and the average Ct was recorded.

### Detection of the breakpoints

A single DNA fragment spanning both breakpoints of the microdeletion was PCR amplified from the mutant allele of proband F13-2 using primer pairs of Forward-Del (5’-GGGATTTGCCTGCTAGCTGAA-3’) and Reverse-Del (5’-AGGGGAAGAGCGAACGGAACT-3’). The breakpoints were determined by Sanger sequencing. PCR amplification of the 1.2-kb product was subsequently used to detect the microdeletion in other family members and 200 ethnically matched normal hearing controls.

## Results

### Clinical characteristics

The affected individuals of Family F13 exhibited typical *NOG*-SSD symptoms including congenital conductive hearing loss (Fig. 1B), proximal symphalangism in the fifth fingers (Fig. 1C), small palpebral fissures, broadened hemi-cylindrical nose with bulbous tip, amblyopia and strabismus. Stapedotomy were performed in subjects F13-2, F13-3, F13-5 and F13-7 and operation identified stapes ankylosis in all. None of the five affected individuals exhibited ID or any other developmental abnormalities.

### Mutation screening of candidate genes

Sanger sequencing in the affected family members identified no pathogenic mutation in the exons and the flanking introns of the candidate genes *NOG*, *GDF5* and *FGF9*. Genotyping of five SNPs within 150 kb in distance to *NOG*, however, showed loss of heterozygosity in the affected family members (Fig. 1A), suggesting the presence of a microdeletion.

### Mapping and determination of the breakpoints

**Fig 2:**
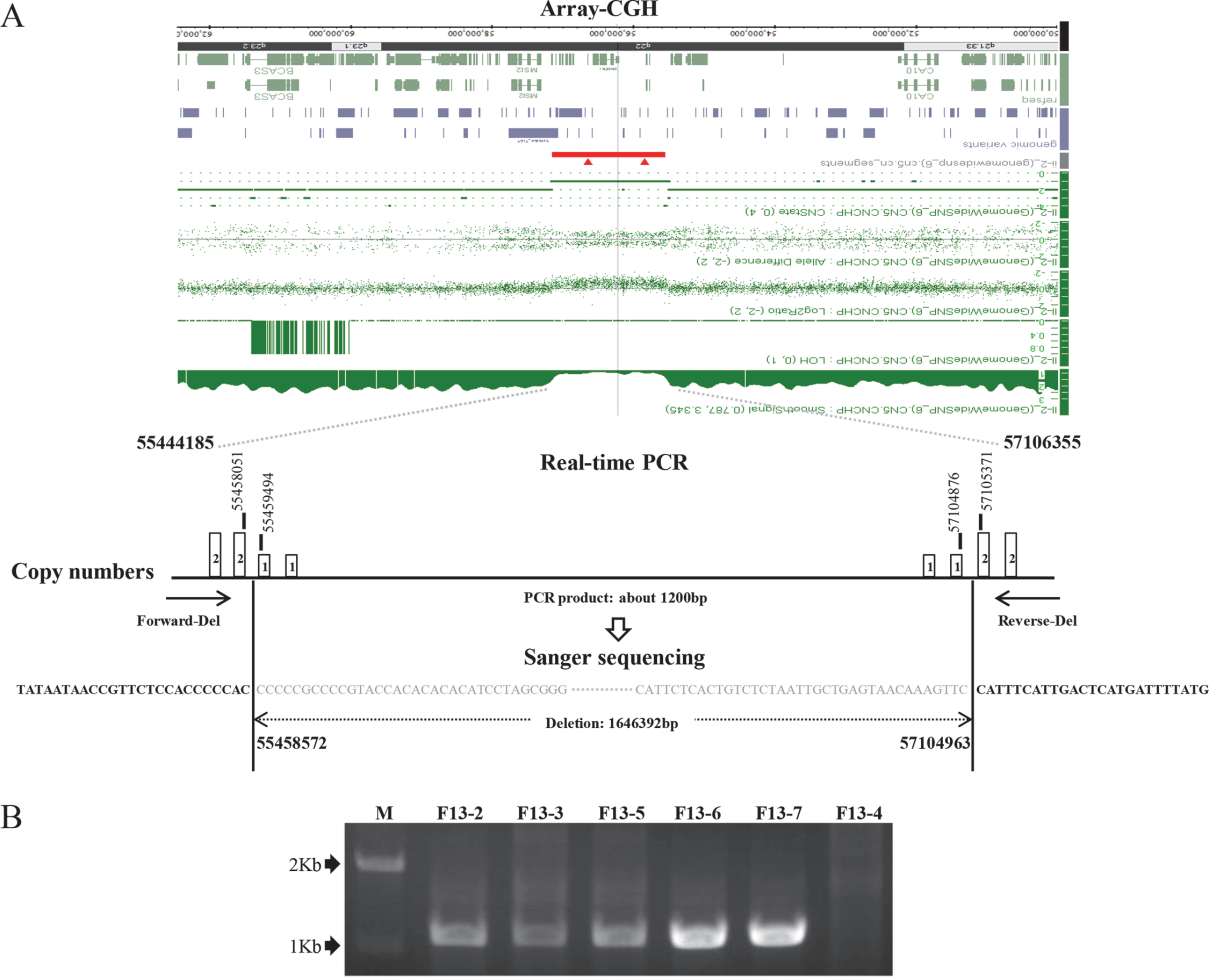
Mapping and identification of the breakpoints of the 1.6-Mb microdeletion in Family F13. A) Schematic illustration of the mapping and identification process. The results of array-CGH, quantitative real-time PCR and sequencing across the breakpoints are shown in the top, middle and bottom panels, respectively. B) A 1.2-kb PCR product was amplified from the mutant allele across the breakpoints in the five affected family members F13-2, F13-3, F13-5, F13-6 and F13-7, but not in the unaffected family member F13-4.

Array-CGH was performed in the proband F13-2 to map the breakpoints of the microdeletion. Single copy losses were observed in an approximate 1.6-Mb genomic region in chromosome 17q22. The maximum deleted region was between 55444185 and 57106355 bases in chromosome 17 (GRCh 38.0) including *NOG* and 12 other genes (Fig. 2A). The positions of the breakpoints were further refined by quantitative real-time PCR to between 55458051 and 55459494 bases on the 5’ side and to between 57104876 and 57105371 bases on the 3’ side. To determine the exact breakpoints, the DNA fragment spanning both breakpoints of the microdeletion was PCR amplified from the mutant allele of F13-2. Sanger sequencing revealed that the microdeletion was from 55458572 to 57104963 bases in chromosome 17 (GRCh 38.0). This deletion was subsequently identified in all five affected family members but not in the unaffected family member F13-4 (Fig. 2B) or 200 ethnically-matched normal controls (data not shown).

## Discussion

In this study, we identified a novel 1.6-Mb microdeletion in chromosome 17q22 in a dominant Chinese Han Family segregating with *NOG*-SSD. To date, over 10 heterozygous microdeletions in the chromosome 17q22 region have been reported causing the *NOG*-SSD-associated disorders, demonstrating that genomic deletions in chromosome 17q22 may be a relatively frequent cause for *NOG*-SSD. Since heterozygous microdeletion cannot be readily detected by the conventional sequencing-based mutation screening methods, alternative tests such as the LOH analysis should be offered to the *NOG*-SSD patients without pathogenic mutations identified in the candidate genes. In a broader sense, genome-wide analysis using the SNP array may be recommended for patients with multiple congenital anomalies.

All previously reported microdeletions in chromosome 17q22 were associated with ID in addition to *NOG*-SSD. Other major abnormalities, observed in some but not all patients, included developmental delay, vertebral anomalies and deficiencies in the reproductive organs such as micropenis, cryptorchidism, absent uterus and rudimentary vagina [5–10]. On the contrary, the symptoms observed in Family F13 were associated with *NOG*-SSD only. None of the five affected family members showed ID or any other developmental abnormalities.

The 1.6-Mb microdeletion identified in Family F13 is among the smallest ones associated with *NOG*-SSD, resulting in a single copy deletion in thirteen genes—*TMEM100*, *PCTP*, *ANKFN1*, *C17orf67*, *DGKE*, *MTVR2*, *MIR3614*, *TRIM25*, *COIL*, *SCPEP1*, *RNF126P1*, *AKAP1* and *NOG*. Of the twelve genes other than *NOG*, only *DGKE* is associated with human disorders, as the recessive loss-of-function mutations in *DGKE* may result in atypical hemolytic-uremic syndrome characterized with microangiopathic hemolytic anemia, thrombocytopenia and renal failure [12,13]. Haploinsufficiency of *NOG* is clearly the key pathogenic mechanism for *NOG*-SSD, which has been supported by studies of the heterozygous *Nog*-deficient mouse model [14].

**Fig 3:**
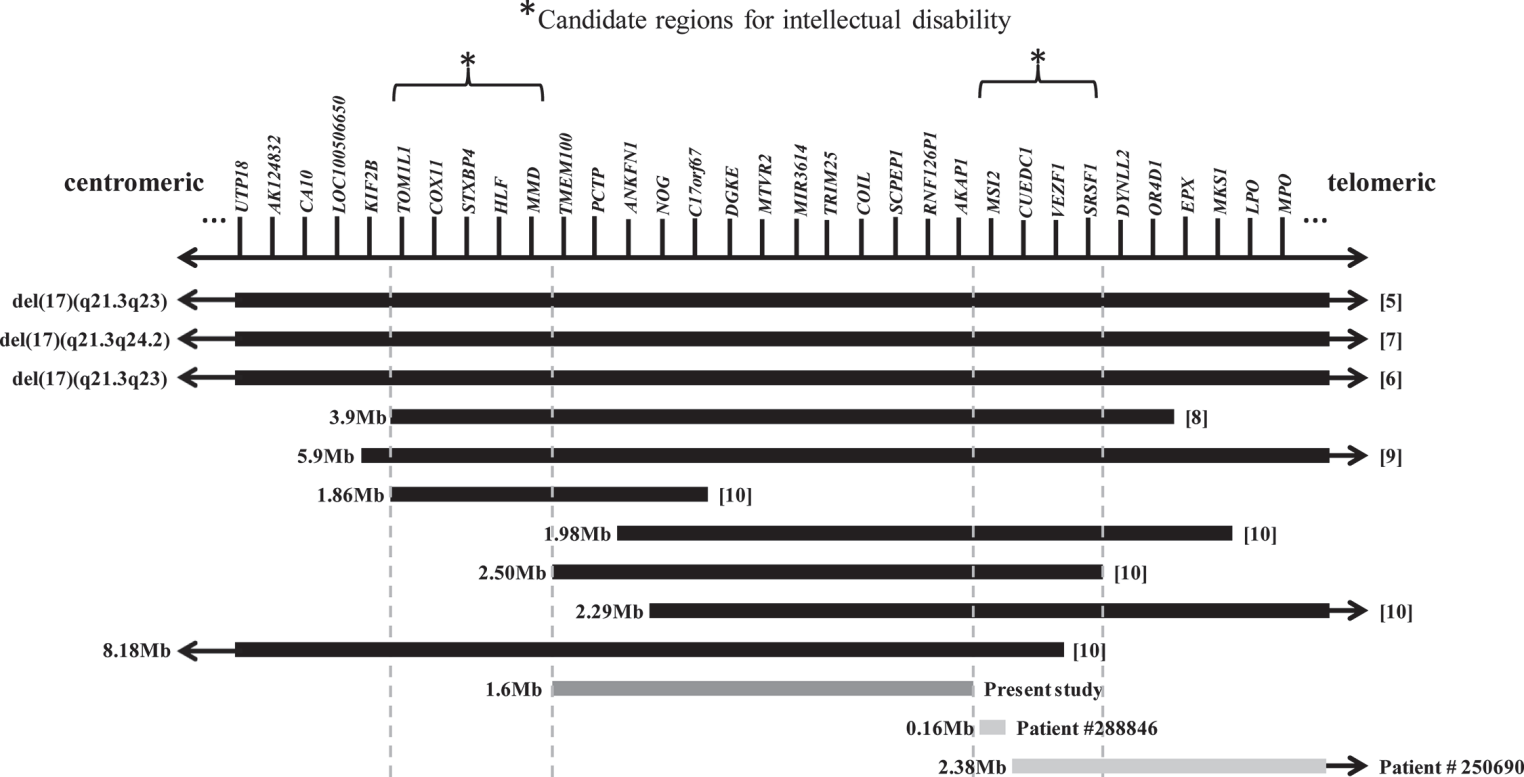
Comparison of the genes and the genomic intervals deleted by the microdeletions in chromosome 17q22 that are associated with *NOG*-SSD only (dark gray), ID only (light gray), or both *NOG*-SSD and ID (black). Candidate regions for causative genes of ID are shown by the brackets on the top.

Since all affected subjects in Family F13 had normal intelligence, it is less likely that the ID reported in other cases was caused by the copy number loss of the thirteen genes or the positional effect of the 1.6-Mb deleted genomic interval. In comparison with the genes and the genomic intervals deleted by other microdeletions in chromosome 17q22, our results may help to narrow down the candidate intervals for ID. As shown in Fig. 3, a 1.86-Mb microdeletion is overlapped with the 1.6-Mb microdeletion on the centromeric side, defining a new candidate interval including *TOM1L1*, *COX11*, *STXBP4*, *HLF* and *MMD*. Similarly, another candidate interval is present on the telomeric side including *MSI2*, *CUEDC1*, *VEZF1* and *SRSF1*. Interestingly, searching of the DECIPHER database (Database of Genomic variants and Phenotype in Humans Using Ensembl Resources, http://decipher.sanger.ac.uk) identified two additional patients with ID but without *NOG*-SSD, who have genomic microdeletions within (patient #288846) or overlapping with (patient #250690) the telomeric candidate interval. These mapping data supported *MSI2* (patient #288846), *CUEDC1*, *VEZF1* and *SRSF1* (patient #250690) as positional candidate genes for ID (Fig. 3).

Searching of the Unigene database (http://www.ncbi.nlm.nih.gov/unigene) showed that all nine candidate genes in the refined intervals are expressed in the brain. *HLF* and *MSI2* in the centromeric and telomeric candidate interval, respectively, are of particular interest based on their function. *HLF* encodes Hepatic leukemia factor, a PAR bZIP (proline and acidic amino acid-rich basic leucine zipper) transcription factor that is increasingly expressed during brain development and plays a role in the function of differentiated neurons [15]. Triple knock-out of *Hlf*, *Tef* and *Dbp*, all three PAR bZIP transcription factor genes, resulted in decreased brain levels of pyridoxal-5-phosphate, serotonin, and dopamine and spontaneous epilepsy in mice [16]. *MSI2* encodes musashi-2, an RNA-binding protein that is concurrently expressed with *MSI1* during development of the central nervous system (CNS) [17]. Down-regulation of both *Msi2* and *Msi1* suppressed the proliferation and maintenance of the CNS stem cells [18]. Overall, our results highlighted several positional candidate genes that could be responsible for the ID phenotypes. Further studies, including both association studies in ID patients and functional studies in the animal models, are needed to elucidate the causative genes for ID in chromosome 17q22.
